# Metformin-Treatment Option for Social Impairment? An Open Clinical Trial to Elucidate the Effects of Metformin Treatment on Steroid Hormones and Social Behavior

**DOI:** 10.3390/life12070998

**Published:** 2022-07-05

**Authors:** Benedikt Gasser, Johann Kurz, Samuel Buerki, Markus Mohaupt

**Affiliations:** 1 Departement für Sport, Bewegung und Gesundheit, University of Basel, 4001 Basel, Switzerland; 2 Interscience Research Association, 8430 Leibnitz, Austria; john@a1.net; 3 Lindenhofspital Teaching Hospital Internal Medicine, 3001 Bern, Switzerland; samuel.buerki@lindenhofgruppe.ch (S.B.); markus.mohaupt@lindenhofgruppe.ch (M.M.)

**Keywords:** GC–MS, urine analysis, androgens, social behavior, androsterone, etiocholanolone, dehydroepiandrosterone, testosterone

## Abstract

Background: Social behavior is mediated by steroid hormones, whereby various lines of evidence indicate that metformin might improve the symptoms of social withdrawal. This directly yields to the aim of the study to correlate the impact of metformin treatment on the potential alterations in steroid hormone homeostasis, which is ultimately impacting social behavior. Therefore, urinary samples of patients before and after treatment with metformin will be correlated to social behavior to elucidate potential changes in steroid hormone profiles and social behavior. Material and Methods: An observational study in healthy adults with a new indication for metformin. Steroid hormone analysis, including the most prominent androgen, estrogen, progesterone, aldosterone, corticosterone, cortisone and cortisol metabolites analyzed with gas chromatography–mass spectrometry and a questionnaire on social behavior (Autism Spectrum Questionnaire (AQ)) will be administered prior to and after around a 12-week phase of metformin treatment. Discussion: It is likely that due to different pathophysiological mechanisms such as an effect on the respiratory chain in mitochondria or via AMP-activated protein kinase, a general alteration of steroid hormone levels can be detected prior to post treatment. The encompassing measurement of steroid hormones shall give hints concerning the involvement of specific cascades yielding potential pharmacological targets for future research.

## 1. Background and Need for This Study

The oral biguanide metformin has been an effective first-line treatment of type 2 diabetes mellitus in adults for more than 60 years. It is to this day the most prescribed oral antiglycemic drug in the world and remains the foundation of type 2 diabetes mellitus treatment, according to the American Diabetes Association, due to its safety, efficacy and cost-efficiency [1,2,3]. Metformin is a valid option when a first increased blood sugar level is detected; however, in the long run, it is not enough for patients suffering from diabetes, and these end up with other pharmaceuticals even with insulin therapy. Nevertheless, recent findings support the acutely decreased hepatic glucose production through a mild transient inhibition of the mitochondrial respiratory chain complex I as the main effect of metformin in reducing blood sugar levels. This reduced hepatic energy state leads to an activation of AMPK (AMP-activated protein kinase), further reducing gluconeogenesis in liver cells. The respiratory chain complex I is further supported as the main target by the preserved metabolic effect of metformin in liver-specific AMPK-deficient mice [4].

Surprisingly, studies have shown metformin treatment reduces macrovascular events and mortality in diabetes mellitus type 2 patients exceeding the expected risk reduction through decreased blood glucose levels. More recent studies have further shown strong support for additional benefits in treating obesity, metabolic syndrome, polycystic ovary disease and depression while also having anti-inflammatory and neuroprotective effects, opening up the possibility for new off-label indications [2,3,4,5].

First, treatment with metformin mimics some of the benefits of caloric restriction, yielding increased insulin sensitivity and reduced low-density lipoprotein and cholesterol levels while inhibiting low-grade inflammation [6,7,8,9,10].

Furthermore, AMP-activated protein kinase activity is increasing antioxidant protection, resulting in reductions in both oxidative damage accumulation and chronic inflammation [6,7,8,9,10,11]. The reduction of oxidative stress, present in several illnesses ranging from cancer up to neurodevelopment disorders with social withdrawal even in their most severe form as autistic diseases [12,13,14], could thus potentially be altered through metformin. To date, the etiopathogenesis or the biochemical basis of autism and neurodegenerative diseases has not been conclusively elucidated, and accordingly, the treatment options are not yet exhausted. Furthermore, we know from analyses of children with autism that there appears to be a change in the cholesterol-dependent steroid metabolism yielding to hyperandrogenism [15,16,17,18]. Causes might be increased oxidative stress resulting in the increased 17,20-lyase-catalyzing activity of adrenal P450c17 through p38α [12]. Interestingly, it was shown that P450c17 phosphorylation selectively increases 17,20-lyase activity and androgen biosynthesis [12]. In consequence, it was indicated that these actions may contribute to the beneficial effects of metformin not only on the reduction of oxidative stress and, in consequence, hyperandrogenism but more generally on the general health and lifespan [9,19]. Direct studies report an effect of metformin on the most famous androgen testosterone in a substantial manner, implying an interdependence of androgens and metformin [20,21]. Furthermore, it was shown that metformin inhibits testosterone-induced stress and unfolded protein response activation by suppressing p38 MAPK phosphorylation (one main cascade in mediating oxidative stress] [22]. These findings can be well embedded in altered kynurenine pathways yielding lower melatonin levels in affected subjects with autism [23,24]. As melatonin is considered to have a very potent antioxidative effect, this allows combining the findings of increased oxidative stress, mitochondrial dysfunction and altered steroid hormones in affected subjects with autism [24]. It was shown in a behavioral mouse model that metformin inhibited the androgen synthesis pathways while also influencing social behavior [19,25]. This might be due to alterations of steroid hormones as studies, for example, pointed to a relationship between testosterone and mainly dominance behavior; however, more recent research suggests that this relationship only holds for individuals with low levels of cortisol, implying that the sum of the metabolites modulates behavior but not the sole concentration of a single metabolite [26]. Furthermore, alterations of steroid hormones would be in line with a dysfunction of the autonomic nervous system (ANS) [27] and a dysregulation of the CRH-ACTH system in autism [28] (reviewed by Taylor and Corbett). This, in addition, directly allows linking steroid hormones via cytokines with inflammation showing the potential broad etiopathogenesis of autism [14].

Additionally, several lines of evidence indicate a beneficial effect of metformin on depression, neurodevelopmental disorders such as autism, neurodegenerative disorders such as Alzheimer’s disease and genetic diseases with neurodevelopmental consequences such as fragile X syndrome [4,29,30]. After only ten days of injection with metformin in a mouse model of Fragile X syndrome (one generally accepted cause of autism spectrum disorders), metformin improved autistic symptoms such as increased grooming and decreased socialization, and treated mice showed normal brain connections and behavioral patterns [31,32,33]. As individuals with autism have social impairments (e.g., a lack of social signal interpretation) and therefore an inability to interpret these signals to guide appropriate behaviors, metformin might be useful as it seems to have a protective effect on social withdrawal potentially mediated by steroid hormones [3,19,30,34,35].

**Figure 1:**
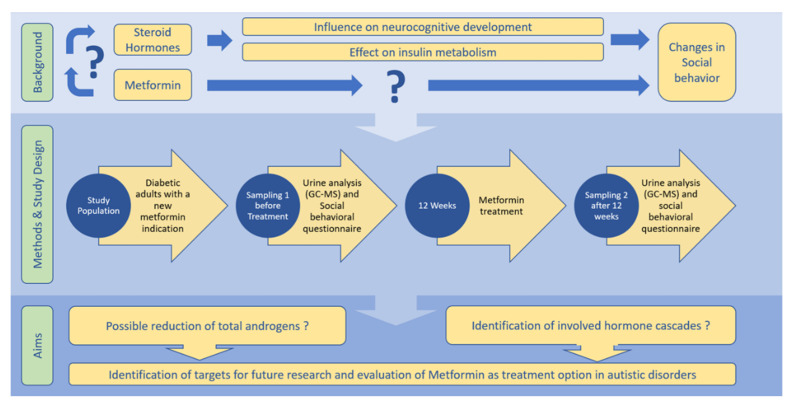
Metformin—Treatment Option for Social Impairment? An Open Clinical Trial to Elucidate the Effects of Metformin Treatment on Steroid Hormones and Social Behavior.

To summarize, the effects of metformin on steroid hormones are, however, only partly elucidated (mainly restricted to the context of PCOS (polycystic ovary syndrome), yielding directly to the aim of this study [2,36,37,38,39,40,41,42,43,44,45].(Figure 1)

## 2. Hypothesis

Several lines of evidence indicate that metformin seems to have an effect on steroid hormones [38,42,46,47,48,49,50]. These studies mainly analyze the effects of metformin on androgen and estrogen markers. Although we already succeeded in showing the involvement of steroid hormones in social withdrawal and interaction with metformin [19], an encompassing analysis opening up possible therapeutic potential is missed. This directly yields to the aim of the study: to assess the effects of metformin on steroid hormones and social behavior. Therefore, we aim to measure steroid hormones from urine samples prior to and during treatment with metformin. Furthermore, we would also like to analyze the effects of metformin on social behavior; therefore, we intend to administer a questionnaire on social behavior to correlate potential alterations in steroid hormones and social behavior.

### 2.1. Primary Research Hypothesis

As a hypothesis with potential falsification, it shall be stated that a newly administered standard therapy with metformin has no influence on steroid hormone profiles in subjects having an indication for Metformin [38,42,46,47,48,49,50,51].

### 2.2. Secondary Research Hypothesis

As a hypothesis with potential falsification, it shall be stated that a newly administered standard therapy with metformin has no influence on social behavior in subjects having an indication for metformin [19,51].

There is no statistical correlation between steroid hormone levels and social behavioral changes [51].

## 3. Material and Methods

### 3.1. Trial Design

As we try to extend our research into the effects of metformin treatment not previously investigated (steroid hormone levels and social behavior), we intend to choose a simple study design.

The study is designed as a clinical trial to investigate changes primary in steroid hormones and secondary in social behavior during metformin treatment. Therefore, a baseline data set will be obtained prior to intervention, and a follow-up at 12 weeks will be conducted. An accompanying social environment anamnesis will also be collected.

The metformin indication itself is independent of study participation and is made following up-to-date diabetes guidelines. The study participation consists of urinary samples and a standardized Autism-Spectrum Quotient questionnaire obtained twice. All patient samples and data will be collected with prior informed consent.

### 3.2. Objectives

#### 3.2.1. Primary Research Objective

This study aims to analyze the effect of metformin treatment on steroid hormone profiles. Therefore, steroid hormones in patients with a new indication for metformin shall be measured with an encompassing gas chromatography–mass spectrometry (GC–MS) prior to treatment start and after 12 weeks of treatment.

#### 3.2.2. Secondary Research Objective

This study will investigate changes in social behavior under metformin treatment. Additionally, to the above-mentioned measurements, an autism questionnaire (Autism-Spectrum Quotient) [52,53,54] shall be conducted at both times. This questionnaire was chosen primarily due to its reliability, objectivity and validity in rapidly assessing the individual traits of an adult in the continuum from normality to autistic behavior, and additionally, the composer of the questionnaire Simon Baron-Cohen is directly addressing steroid hormones in his research [55].

This study will elucidate the potential correlation between steroid hormone levels and social behavioral changes.

### 3.3. Endpoints

#### 3.3.1. Primary Research Endpoint

Changes in steroid hormone profiles of interest (see below) prior to metformin treatment start and after 12 weeks of treatment.

#### 3.3.2. Secondary Research Endpoint

Changes in social behavior measured by the Autism-Spectrum Quotient AQ (see below) prior to metformin treatment start and after 12 weeks of treatment.

Correlation between steroid hormone profiles and social behavioral changes.

### 3.4. Eligibility Criteria

#### 3.4.1. Inclusion Criteria

Patients (male or female) older than 18 years with type 2 diabetes mellitus and an indication for metformin according to the American Diabetes Association, starting a new metformin treatment or are pausing treatment for example due to a further medical investigation for at least seven days. The main marker will be fasting plasma glucose levels of≥ 7.0mmol/L and/or HbA1c ≥ 6.5% [56].

#### 3.4.2. Exclusion Criteria

- Patients under 18 years of age.
- Clinically significant concomitant disease (e.g., advanced renal failure, hepatic dysfunction, neoplasia).
- Significant musculoskeletal disease.
- Active infection during sample collection.
- Immunosuppressive medical therapy.
- Hormonal/steroid treatment.
- Pregnancy.
- Psychiatric disease and known social-behavior-altering medication (e.g., antipsychotic medication).
- Known or suspected malcompliance, drug or alcohol abuse.
- Inability to follow the procedures of the study, e.g., due to insufficient language skills, severe dementia.
- Life expectancy less than 6 months.
- Poor tolerability to metformin treatment with following treatment discontinuation within duration of follow-up.
- Pharmaceutical treatment with insulin or another pharmaceutical with a known strong effect on blood sugar levels.

### 3.5. Intervention and Follow-Up

In the 1980s, a correlation between insulin, insulin-sensitizing drugs (e.g., metformin) and androgens was already being implied [57]. In studies conducted on women with PCOS, a treatment duration of 6 weeks of metformin at a target dosage (1500mg daily) was deemed appropriate for a substantial effect on hormonal levels, with the results showing significant changes [41]. Therefore, we will be collecting the follow-up samples and data 12 weeks after treatment starts. As the treatment will be conducted by the treating physician, there might be differences between patients in metformin dosage during the saturation period before reaching target dosage. The 12-week follow-up will ensure a sufficient treatment time passed. Target dosage (500mg–3g per day) and saturation period will be according to the treating physician. The cumulative dosage received will be quantified for every patient enrolled to allow potential analyses for dosage-related effects (standardized for treatment duration).

To research the primary endpoint of steroid hormone profile changes, we will collect urinary samples prior to metformin treatment start and after 12 weeks of treatment. Similarly, to research the secondary endpoint of social behavioral changes, we will conduct a questionnaire (Autism-Spectrum Quotient) along with social environmental anamnesis at both times.

The additional medical risks of this study are to be assessed as very low (collection of urine samples along with a questionnaire during a patient interview). Risks arise only from the administration of metformin, which has to be assessed as a safe, very well-known and frequently used oral antiglycemic drug, the only severe side effect being lactic acidosis [1]. In general, metformin, which would have also been the first-line treatment without conducting the study, is a sensible choice for the treatment of type 2 diabetes mellitus [1].

### 3.6. Recruitment

As the detailed sample size calculation (see Section 4.4) already indicates to have an effect with a small sample size of seven persons, the aim is in consequence to gain 10 female and male patients of approximately the same age for the study estimate to conduct first rough analyses.

Further, based on the number of patients per year in our clinical setting, it seems reasonable to win enough patients within 2 years. We estimate an average of one patient a week, yielding a total of 50 patients a year so that we will have enrolled a total of around 100 patients after 2 years. The recruitment will be conducted by the department of internal medicine of the Lindenhofgruppe and its associated General practitioners. Due to the common characteristics of patients with type 2 diabetes mellitus we expect to mainly recruit patients above 50 years of age and slightly more male than female patients. Furthermore, the obstetrics department of Inselspital Berne with its Patients with a potential indication of metformin due to PCOS will also be included.

## 4. Data Collection Methods

### 4.1. Steroid Measurement Procedures

Analysis of urinary steroids will be conducted via gas chromatography–mass spectrometry. Urine samples shall be taken prior to treatment start and after 12 weeks of treatment (gold standard: 15 mL from a 24 h urine collection). Urine sample preparation comprises pre-extraction, enzymatic hydrolysis, extraction from the hydrolysis mixture, derivatization and gel filtration. The recovery standard will be prepared by adding 2.5 µg of medroxyprogesterone to 1.5 mL of urine. The sample will be extracted on a Sep-Pak C18 column (Waters Corp., Milford, MA, USA), dried, reconstituted in a 0.1 M acetate buffer, pH 4.6, and hydrolyzed with a powdered Helix pomatia enzyme (12.5 mg; Sigma Chemical Co., St. Louis, MI, USA) and 12.5 µL of β-glucuronidase/arylsulfatase liquid enzyme (Roche Diagnostics, Rotkreuz, Switzerland). The resulting free steroids will be extracted on a Sep-Pak C18 cartridge. A mixture of internal standards (2.5 µg each of 5α-androstane-3α, 17α-diol, stigmasterol, and cholesterol butyrate, and 0.15 µg of 3β5β-tetrahydroaldosterone) will be added to this extract, and the sample will be derivatized to form the methyloxime-trimethylsilylethers. Analyses will be performed on a Hewlett Packard gas chromatograph 6890 (Hewlett Packard, Palo Alto, CA, USA) with a mass selective detector 5973 by selective ion monitoring (SIM). One characteristic ion will be chosen for each compound measured. The derivatized samples will be analyzed during a temperature-programmed run (210–265 °C) over a 35 min period. The calibration standard consists of a steroid mixture containing known quantities of all the steroid metabolites to be measured. Responses and retention times will be recorded regularly. In each case, the ion peak will be quantified against the internal stigmasterol standard. All procedures will be performed as described several times by us and others [16,18,58,59,60].

The following metabolites shall be measured as described above [15,16]:

Androgen metabolites: Androsterone, Etiocholanolone, Androstenediol, 11-Oxoetiocholanolone, 11β-Hydroxyandrosterone, 11β-Hydroxyetiocholanolone, Dehydroepiandrosterone, 5-Androstene-3β,17β-diol, 16α-Hydroxydehydroepiandrosterone, 5-Androstene-3β,16α,17β-triol, 5-Pregnene-3β, 16α,17β-triol, Testosterone, 5α-Dihydrotestosterone

Estrogen metabolites: Estriol, 17b-Estradiol.

Progesterone metabolites: 17-Hydroxypregnanolon, Pregnanediol, Pregnanetriol, 11-Oxo-Pregnanetriol Aldosterone-Metabolites: Tetrahydroaldosterone.

Corticosterone metabolites: TetrahydroDOC, Tetrahydrodehydrocorticosteron, Tetrahydrocorticosteron, 5a-Tetrahydrocorticosteron, 18-Hydroxy-tetrahydrocompound A, Cortisone.

Cortisone metabolites: Tetrahydrocortisone, a-Cortolon, b-Cortolon, 20a-Dihydrocortison, 20b-Dihydrocortison.

Cortisol metabolites: Cortisol, Tetrahydrocortisol, 5a-Tetrahydrocortisol, a-Cortol, b-Cortol, 20a-Dihydrocortisol, 6b-Hydroxycortisol, 18-Hydroxycortisol.

### 4.2. Social Behavior Measurement Methods

In order to measure social behavioral changes related to typical behaviors of autism and expand the idea of autism being related to steroid hormone levels, we will be conducting an autism questionnaire prior to and after 12 weeks of treatment. The Autism Spectrum Quotient (AQ) is a questionnaire published in 2001 by Simon Baron-Cohen and his colleagues at the Autism Research Center in Cambridge, UK [52,53,54]. As later research by this group was yielding to steroid hormones and due to its simplicity, we consider this questionnaire as the right choice. Consisting of 50 questions, the aim is to investigate whether adults of average intelligence have traits of autistic behavior. We will analyze the questionnaire according to the guidelines developed by Baron-Cohen et al., 2001.

### 4.3. Data Collection, Management and Retention

Data will be anonymized and stored in a pseudonymized manner. For females before menopause, the day of the menstrual cycle will be reported for further analyses. Personal data including ethnicity will be entered into a separate Excel file with restricted access only by the core staff of the study. Collection and management of the clinical trial data will be done using an electronic data capture system (Red Cap Cloud). Source data will be kept under lock and key. Password protection ensures that only authorized persons can enter the system to view, add or edit data according to their permissions. The complete study dataset is exported and transferred to the investigators as well as to the study statistician through a secured channel. Data will be archived at the Lindenhofspital. Independently from investigators, regulatory authorities can audit this trial. Direct access to source documents will be permitted for purposes of monitoring, audits and inspections. All involved parties must keep the participant’s data strictly confidential. Any results of this study will be published in an anonymized manner. The study protocol and dataset shall be accessible to any regulatory authority after publication for at least 10 years.

### 4.4. Sample Size

Per Morin-Papunen et al., 2003, the most prominent serum metabolite testosterone decreased by 2.7 ± 0.3 to 2.0 ± 0.2 nmol/liter in patients receiving metformin (500 mg twice daily for 3 months or longer, then 1000 mg twice daily for 3 months or longer) [61]. By taking an alpha of 0.05% corrected by Bonferonni (0.005/44 (number of metabolites intended to analyze) = 0.001136) and a target power of 80% we would get an n = 7 patients [62]. However, this must be taken with a grain of salt as a very optimistic assumption for our purposes. We suggest that for not yet measured and reported effects on metabolites such as, e.g., androsterone, eticholanolone, dehydroepiandrosterone, epitestosterone effect sizes are much lower in the range of one-fourth of the above-mentioned testosterone. Therefore, the number needed to analyze will be around 45 patients. This estimate would be further supported by analyses by Baillargeon et al., 2004 [42], Chou et al., 2003 [46], Cibula et al., 2005 [47], Gambineri et al., 2006 [48], Ibanez et al., 2004 [49], Nestler et al., 1997 [41] and Tang et al., 2006 [50] analyzing effects of metformin mainly on androgen and estrogen markers. Furthermore, the proposed number of 45 would also potentially allow us to find an effect on steroid hormone profiles at lower dosages than described by Morin-Papunen [61]. Concerning the Autism questionnaire, it is to mention that an average score in students of 17.6 ± 6.4 was detected compared to healthy controls with an average score of 16.4 ± 6.3, yielding an alpha of 0.05% and a target power of 80% to a large sample size of 894 subjects [53]. When performing a comparison with students participating in the Math Olympiad scoring 24.5 ± 6.4 versus students with the already mentioned score of 17.6 ± 6.4, a sample size of 28 results [53]. These findings do not differ largely when it is stratified for sex, despite the fact that women tend to score lower [38]. To summarize, if there is a substantial effect of metformin, the proposed sample size of around 45 will be sufficient to detect differences.

## 5. Statistical Analysis

Mean and SEM (standard error of mean) of all metabolites will be calculated pre-versus post intervention for the respective sub-samples distinguished by age and sex. To analyze the distribution patterns of the measured values of each metabolite Kolmogorov–Smirnov tests will be conducted. If normality distribution is present, the differences between pre-versus post intervention shall be analyzed with two-tailed dependent t-tests while correcting for multiple comparisons with Bonferroni correction. If the hypothesis of normal distribution must be rejected for metabolite concentrations pre-versus post intervention, Wilcoxon tests shall be conducted. If normal distribution is present, differences pre-versus post intervention will be further quantified with the calculation of effect sizes by Cohen with pooled standard deviation and 95% confidence intervals [63,64]. For metabolites not showing a normal distribution, effect sizes shall be calculated according to Pallant (2005) [65,66]. Correlation analysis (Pearson correlation coefficient) will be conducted to detect a potential relationship between cumulative metformin dosage (standardized for body weight and treatment duration] and steroid hormone concentrations, respectively, scores from the autism questionnaire. For female subjects before versus after menopause, analyses will be conducted separately. Potential differences will be compared with prior gained reference values from our lab [67]. As we do have a pool of healthy subjects, an individually pairwise matched design (twin matching) might be another possibility to reduce an age bias increasing the validity of the results [67].

Based on these preliminary analyses, structure equation modeling can be applied if indicated in order to combine findings from biochemical analysis and from questionnaires. All calculations will be performed with GraphPad Prism (GraphPad Software, Inc., La Jolla, CA, USA) and SPSS (Microsoft Inc., Redmond, WA, USA).

## 6. Discussion

The aim of this study is to analyze the effects of metformin treatment on steroid hormone profiles. To the best of our knowledge, there is no study directly elucidating the presumed effects on steroid hormones and social behavior in humans [19]. The description of potential steroid hormone alterations will allow making different statements concerning pathway activities such as, for example, enzymes involved. However, we will not be able to understand the complexity of all steroid hormone pathways [68]. Characterizing the ligands will allow to descriptively develop an understanding; however, as ligand binding induces specific conformational changes in the ligand-binding domain, which can modulate surface topology and protein-protein interactions between androgen receptors and coregulators, resulting tissue-specific gene regulation processes cannot be fully captured with this descriptively oriented analysis [68].

We are aware that the optimal study design would be a placebo-controlled crossover trial, which is, however, for practical reasons, impossible to conduct. Nevertheless, several lines of evidence suggested the involvement of steroid hormones in autism, especially increased androgens [15]. Just recently, a meta-analysis and systematic review performed by us showed higher androgen levels in hand with increased 17/20 lyase activity in children with autism [66]. Furthermore, the involvement of glucose homeostasis factors such as insulin and growth hormone is likely [69]. Males with Asperger’s syndrome showed altered levels of 24 biomarkers, including increased levels of cytokines and other inflammatory molecules [69]. We know from our own analyses of children with autism spectrum disorders that those affected show a substantial change in steroid hormone profiles [16,18]. In our mice model, we were able to show a change in social behavioral patterns, including social withdrawal, after receiving metformin treatment [19]. These findings are to be expanded with a clinical focus and tested in humans. Thereby, as steroid hormones may exert different effects on the brain, respectively cognitive functions such as learning, emotional judgment or face recognition, the findings might have a high value from a translational point of view [70,71].

For this purpose, we want to analyze the change in social behavior and steroid hormone profiles, as well as their potential correlation, under metformin treatment. Because only urinary metabolites of steroid hormones and not the blood serum levels themselves will be examined, there is a certain limitation concerning the reliability of measured outcomes; also, comparisons with other studies might be restricted. We chose this measurement because of its general availability and ease of use for both patient and physician to ensure easier recruitment and follow-up. As a general research question, we suspect that certain substances modulate social behavior via steroid hormones, which act as an intermediary in social behavior. Therefore, we will measure both urinary steroid hormone profiles as well as social behavior through an Autism Spectrum Quotient respectively questionnaire before treatment start and after.

Although we expect our patient sample of adults not typically affected by social behavioral disorders or autism spectrum disorders to score in the lower spectrum of the questionnaire, we have chosen this tool because of its simplicity in use to ensure sufficient patient recruitment and follow-up. The questionnaire has been tested in both adults with autism spectrum diseases as well as healthy adults. In healthy adults, significant differences between genders and different backgrounds (mathematics vs. humanities) were recorded, while all scores remained in the lower spectrum [53]. As discussed above, we expect social behavioral changes to impact foremost social withdrawal and anxiety, traits that are well covered in the AQ. Thus, we hope to record significant changes even in healthy adults. Furthermore, we already showed an effect in our own mouse model while also using a strong simplification of social behavioral measurement [19]. Yet, the simple way to assess social behavior remains one limitation; more proven and elaborative possibilities might be used, especially in studies explicitly focused on specific pharmaceutical targets and their involvement in social behavior.

Regarding patient recruitment, we are well aware of potential effect alterations due to their type 2 diabetes mellitus, the difference in sex and baseline hormone levels (especially in females pre-versus postmenopausal), as well as an expected higher age. We chose this patient setting because we wanted to be able to recruit enough patients in a relatively short period while also ensuring safety for the participants, as they all would have received the metformin treatment without our study anyway. This patient collective also shares many characteristics with a majority of cancer patients who are also often severely impacted by social impairment due to their disease.

Nevertheless, we hope to show the influence of metformin treatment on social behavior and/or steroid hormone profiles. Published in a recognized specialist journal, these findings could trigger further research within a more specific patient setting of social avoidant people in a randomized, double-blind placebo-controlled study.

If we do succeed in showing a correlation between social behavior and steroid hormone profile changes under metformin treatment, this could result in a recommendation for further, more focused research examining the cascades of metformin’s effects even further and the possibility to influence more biochemical cascades will arise. A better insight into the effects of one of the most common drugs worldwide may result in more specific and targeted treatment, maybe becoming a part of personalized medicine.

## 7. Ethics and Dissemination Policy

### 7.1. Ethics and Dissemination

The study is in the lowest class A (per the ethics committee, see below). It is a therapeutic exploratory study per ICH E8 [72]. This study will be conducted in compliance with the Declaration of Helsinki, the ICH-GCP or ISO EN 14155, as well as all national legal and regulatory requirements.

According to the legal situation in Switzerland, the study is considered within the Humanforschungsgesetz (HFG) [73] and Verordnung über die Humanforschung (HFV) [74]. The study risks seem very low, as only measurements of urine and the administration of a questionnaire are executed, resulting in Category A per HFV.

### 7.2. Trial Status

The trial will be conducted per protocol version 1.0. Patient recruitment will start in Winter 2021 and is expected to run consecutively until the end of December 2023. At the time of submission, zero patients have been included in the study. Data collection is expected to be completed in December 2023 and data analysis in Spring 2024.

### 7.3. Ethics Approval and Consent to Participate

Ethics approval to conduct this trial has been granted by the local Ethics Committee for the Region of Bern (Project-ID: 2020-02913). The trial will meet the criteria and principles of the Declaration of Helsinki and has been registered in the Clinicaltrials.gov database (Trial registration number: NCT04930471, Registered 17 June 2021).

Informed consent to participate in the trial will be obtained by the study physician from all patients prior to entry into the study. Each patient will be informed that participation in the study is voluntary and that he/she may withdraw from the study at any time with no need for justification, and that withdrawal of consent will not affect his/her subsequent medical assistance and treatment. Furthermore, the patient will be informed on an obligatory basis that his/her medical records may be examined by authorized individuals other than their treating physician. All patients are covered by liability insurance for the total study duration.
